# Roles and Clinical Applications of OPG and TRAIL as Biomarkers in Cardiovascular Disease

**DOI:** 10.1155/2016/1752854

**Published:** 2016-04-21

**Authors:** Stella Bernardi, Fleur Bossi, Barbara Toffoli, Bruno Fabris

**Affiliations:** Department of Medical, Surgical and Health Sciences, University of Trieste, Cattinara Teaching Hospital, Strada di Fiume, 34149 Trieste, Italy

## Abstract

Cardiovascular diseases (CVD) remain the major cause of death and premature disability in Western societies. Assessing the risk of CVD is an important aspect in clinical decision-making. Among the growing number of molecules that are studied for their potential utility as CVD biomarkers, a lot of attention has been focused on osteoprotegerin (OPG) and its ligands, which are receptor activator of nuclear factor *κ*B ligand (RANKL) and TNF-related apoptosis-inducing ligand. Based on the existing literature and on our experience in this field, here we review what the possible roles of OPG and TRAIL in CVD are and their potential utility as CVD biomarkers.

## 1. Introduction

Cardiovascular diseases (CVD) remain the major cause of death and premature disability in Western societies. In 2013 there were more than 54 million deaths globally and 32% of them (17 million) were attributable to CVD [1]. Moreover, current predictions estimate that by the year 2020 cardiovascular diseases, notably atherosclerosis, will become the leading global cause of total disease burden [2]. These figures reinforce the need for diagnostic-prognostic tools that could help identify the subset of patients with the highest risk of morbidity and mortality from CVD and, therefore, that could help better tailor/focus our interventions.

Among the growing number of molecules that are studied for their potential utility as CVD biomarkers, much attention has been focused on osteoprotegerin (OPG) and its ligands, which are receptor activator of nuclear factor kB ligand (RANKL) and TNF-related apoptosis-inducing ligand (TRAIL), as reviewed in [3–6]. OPG is in fact a circulating glycoprotein, which was first characterized for its ability to block RANKL and inhibit bone reabsorption, hence its name. Subsequently, it has been demonstrated that OPG can inhibit TRAIL peripheral actions, which are related to cellular life and death, and that it can also have direct (ligand-independent) effects on the bone, the vasculature, and the immune system.

While the significance of OPG for vascular biology has gained epidemiological support [7], with a range of studies reporting associations between circulating OPG and incident CVD [8–10], the role and significance of RANKL and TRAIL are less clear. Recently, Secchiero and colleagues reported that patients with coronary artery disease displayed an increased OPG/TRAIL ratio, which was even higher in the subgroup of patients who developed heart failure, thus suggesting that the OPG/TRAIL ratio plays a significant role in the pathophysiology of CVD [11]. Here we review what the possible roles of OPG and TRAIL in CVD are and their potential utility as CVD biomarkers.

## 2. Overview on OPG and TRAIL Biology

### 2.1. OPG Biology

**Figure 1:**
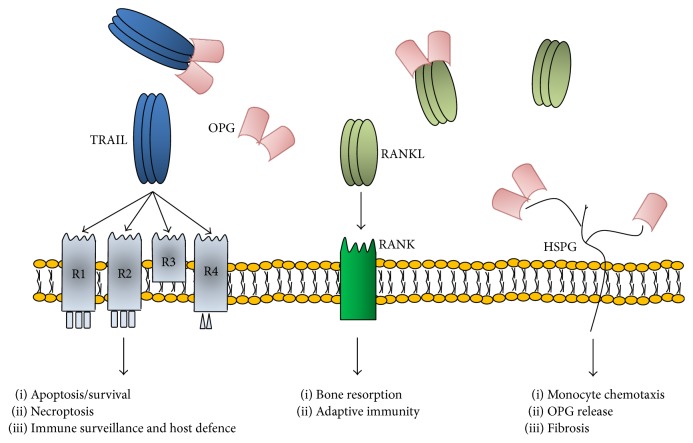
Representation of the TRAIL/OPG/RANKL system. Osteoprotegerin (OPG) is a secreted glycoprotein, whose predominant and more bioactive extracellular form is a disulphide-linked dimer. By acting as a decoy receptor for TRAIL and RANKL, OPG regulates many processes, such as cell apoptosis/survival and necroptosis, immune surveillance and host defence, and bone resorption. Moreover, OPG binds glycosaminoglycans such as heparin sulfate proteoglycans (HSPG), whereby it regulates monocyte chemotaxis, OPG release, and fibrosis. As for TRAIL, it is expressed as a transmembrane protein, which can be cleaved and released as a soluble molecule, which combines with two other molecules of TRAIL to form a trimeric ligand. TRAIL homotrimers bind to their specific receptors, which include two death receptors, TRAIL-R1 and TRAIL-R2, and three decoy receptors, TRAIL-R3, TRAIL-R4, and osteoprotegerin (OPG). Likewise, RANKL can be found in both membrane-bound and soluble forms. When it is released as a soluble molecule, RANKL combines with two other molecules of RANKL to form a trimeric ligand, which binds to its receptor RANK. HSPG is heparin sulfate proteoglycans; OPG is osteoprotegerin; R is receptor; RANK is receptor activator of nuclear factor kappa-B, RANKL is receptor activator of nuclear factor kappa-B ligand; TRAIL is TNF-related apoptosis-inducing ligand.

Osteoprotegerin (OPG) is a protein that belongs to the tumor necrosis factor (TNF) superfamily, which was identified by three independent groups [12–14]. Following the observation that when this molecule was injected into mice it increased their bone mass [15], the American Society of Bone and Mineral Research Committee called it osteoprotegerin [16] because it described its bone protective actions. In humans, OPG is expressed in health and disease states in a wide variety of tissues [3]. These include not only the bone [17–19], but also the heart, vessels, kidney, liver, spleen, thymus, lymph nodes [20], as well as the adipose tissue, and pancreas [21–23]. In the vessels, OPG is expressed by endothelial [24] and smooth muscle [25] cells. The gene encoding for OPG is located on chromosome 8 at position 8q24 [12], in a region that seems to harbor a gene cluster involved in the regulation of bone development and metabolism [12]. OPG gene locus spans approximately 29 kb and it has five exonic segments. OPG is expressed as a circulating glycoprotein of 401 amino acids with seven structural domains. Among them, domain 7 contains a heparin-binding region as well as the free cysteine residue that is required for disulphide bond formation and allows OPG to interact and combine with another molecule of OPG to form a dimeric ligand [12]. Therefore, circulating OPG can be found either as a free monomer of 60 kD or as a disulphide bond-linked homodimer form of 120 kD, which is usually biologically more active than the monomeric one [12]. Moreover, OPG can also circulate while bound to its ligands, which are RANKL and TRAIL, as represented in Figure 1.

RANKL and TRAIL are also two members of the TNFR superfamily of proteins that, in the absence of OPG, usually bind to specific transmembrane receptors and activate downstream signaling. On the one hand, by blocking RANKL [26], which stimulates osteoclast formation and activation [27], OPG prevents bone loss; this represents the rationale for its current use in patients with osteoporosis [28, 29]. On the other hand, by blocking TRAIL, OPG prevents TRAIL-induced apoptosis of tumor cells [30]. However, given that TRAIL induces apoptosis in transformed cells such as malignant, virally infected, and overactivated cells, while it spares the normal ones, the actions of TRAIL (and therefore of OPG-TRAIL) are less well characterized in nontransformed cells. Moreover, OPG may also have direct (ligand-independent) actions in the vasculature, bone, and immune system, mediated by its heparin-binding domain [31–33], which interacts with cellular heparin sulfate proteoglycans that usually take part in cell-surface signaling [34].

**Figure 2:**
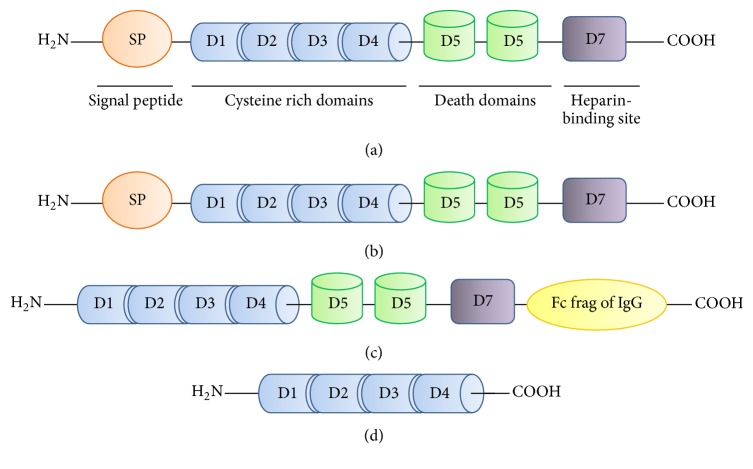
Schematic representation of OPG structural domains as compared to the standards of the available ELISA kits. (a) OPG structural domains; (b) R&D Duoset ELISA standard; (c) BioVendor ELISA standard; (d) Biomedica ELISA standard. ELISA is for enzyme-linked immunosorbent assays; OPG is for osteoprotegerin.

It has to be noted that current enzyme-linked immunosorbent assays (ELISA) measuring circulating OPG do not differentiate between its form (monomer rather than disulphide-linked dimer) and site of origin [6]. Moreover, OPG can be quantified by different ELISA (R&D Duoset, BioVendor, and Biomedica) [6], which use different forms of the molecule as the reference standards (Figure 2). This results in differences in the lower detection limits (being 65 pg/mL for R&D Duoset, 115 pg/mL for BioVendor, and 1.4 pg/mL for Biomedica) as well as in the final concentrations [35]. Clancy and colleagues [36] demonstrated that OPG concentrations for the same samples were significantly different when they were measured by different assays, while concordance correlation coefficients for intra- and interassay reproducibility were good.

### 2.2. TRAIL Biology

As mentioned earlier, TRAIL is also a protein that belongs to the TNF superfamily and was cloned on the basis of its high homology to other TNF family members, such as FasL/CD95L and TNF-*α* [37]. The percentage of identity with FasL/CD95L and TNF-*α* is in fact 28% and 23%, respectively. In humans, TRAIL is expressed in health and disease states in a wide variety of tissues, including the vessels, where it is expressed in vascular smooth muscle cells (VSMC) [38]. The gene encoding for TRAIL is located on chromosome 3 at position 3q26. TRAIL gene locus spans approximately 20 kb and it has five exonic segments. In humans, TRAIL is expressed as a type II transmembrane protein of 281 amino acids. Like TNF-*α*, TRAIL can be cleaved at the stalk domain, and by combining with other two molecules of TRAIL, it forms a circulating homotrimer with biological activity [39]. As represented in Figure 1, the human receptors for TRAIL include not only death receptors (DR) but also decoy receptors (DcR) [40, 41]. TRAIL DR comprise TRAIL-R1 [42] and TRAIL-R2 [43], which are both type I transmembrane proteins containing an intracellular death domain (DD) that classically stimulates apoptosis upon TRAIL binding and are both expressed in the vessels. Compared to TRAIL, which is normally expressed by VSMC, TRAIL-R1 and TRAIL-R2 are also expressed by endothelial cells (EC) [44–46]. As for TRAIL DcR, they include TRAIL-R3 [47], TRAIL-R4 [48, 49], and OPG [50]. DcR1 and DcR2 are transmembrane receptors that differ from DR in that their cytoplasmatic domain lacks an intact DD, while OPG is a soluble decoy receptor that is lacking both transmembrane and cytoplasmatic residues.

In the absence of OPG, TRAIL homotrimers bind TRAIL-R1 and TRAIL-R2 on the surface of target cells (Figure 1). Through such binding, TRAIL is able to trigger cellular apoptosis in malignant, virally infected, and overactivated immune cells, hence its acronym. Recently, it has been shown that TRAIL can also induce necroptosis, which is a regulated and programmed form of necrosis that takes place after TRAIL binding to its specific death receptors and which can be useful to the body when apoptosis has been blocked [51, 52]. With respect to TRAIL's ability to induce apoptosis in tumor cells, studies on TRAIL-knockout mice have in fact demonstrated that mice without TRAIL are viable and fertile but more susceptible to tumor metastases, indicating that TRAIL regulates immune surveillance and host defence against tumor initiation and progression [53, 54]. In particular, TRAIL seems to mediate the ability of natural killer cells and cytotoxic T lymphocytes to block tumor growth and metastasis development [55]. Interestingly, one of the unique aspects of TRAIL, as compared to other proapoptotic ligands [56, 57], is that TRAIL has the ability to induce apoptosis preferentially in transformed cells, such as tumor or infected cells, while it spares the normal ones [58]. In particular Ashkenazi and colleagues demonstrated that the exposure of cynomolgus monkeys to recombinant human- (rh-) TRAIL at 0.1-10 mg/Kg/day over 7 days did not induce detectable toxicity, whereas, by comparison, TNF-*α* induced severe toxicity at much lower doses such as 0.003 mg/Kg/day [59]. This is the rationale for its use in clinical settings as an antitumor drug [39].

While it has been clearly demonstrated that TRAIL induces apoptosis in transformed cells, in nontransformed cells, the actions of TRAIL are less well characterized. For example, this molecule could actually mediate nonapoptotic signaling. It has in fact been shown that when TRAIL-R1 and TRAIL-R2 are activated they not only stimulate the extrinsic apoptotic pathway, but also may activate survival/proliferation pathways, such as nuclear factor *κ*B (NF-*κ*B), ERK1/ERK2, and Akt [44, 60] (Figure 1). Consistent with the concept that TRAIL triggers nonapoptotic signals in normal cells, we have also shown that systemic TRAIL delivery significantly reduced cardiac fibrosis and apoptosis in a mouse model of diabetic cardiomyopathy [61]. Potential mechanisms underlying the ability of TRAIL to activate such opposed pathways include the redistribution of TRAIL receptors [62, 63] and the intracellular inhibition of the apoptotic cascade [64].

## 3. Role of OPG and TRAIL on Atherosclerosis 

### 3.1. OPG and Atherosclerosis

**Figure 3:**
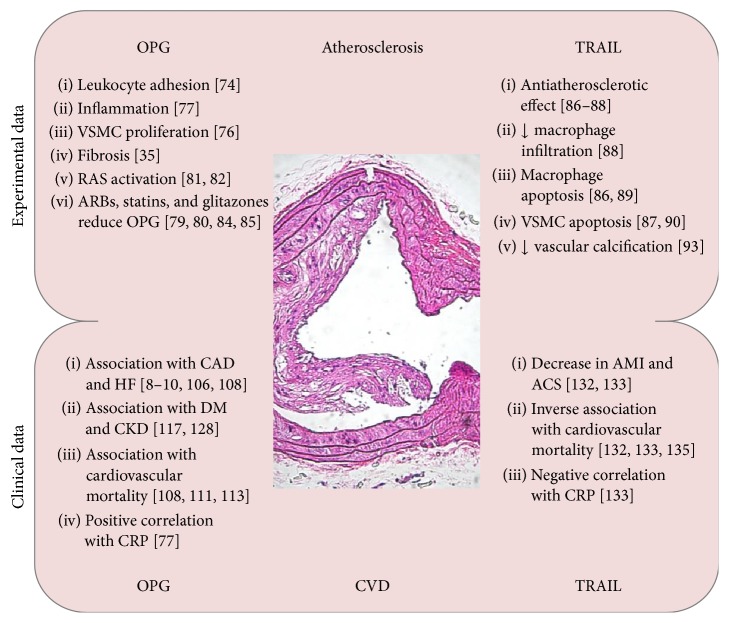
Roles of OPG and TRAIL in atherosclerosis and CVD. In the upper part of the image, summary of the main experimental data supporting OPG and TRAIL involvement in atherosclerosis. In the lower part of the image, summary of the main clinical data showing OPG and TRAIL associations with CVD. In the middle, representative image of an aortic atherosclerotic plaque stained by hematoxylin and eosin (10x original magnification). ACS is acute coronary syndromes; AMI is acute myocardial infarction, and ARBs are angiotensin II type 1 receptor blockers; CAD is coronary artery disease; CKD is chronic kidney disease; CRP is C-reactive protein; DM is diabetes mellitus; OPG is osteoprotegerin; RAS is renin-angiotensin system; TRAIL is TNF-related apoptosis-inducing ligand; VSMC is vascular smooth muscle cell.

The current view of atherosclerosis is that it is an inflammatory disease of the vessels [65], mediated by leukocyte vascular recruitment and migration. In particular, once different stimuli/forms of injury increase endothelium adhesiveness to circulating cells, leukocytes migrate into the subendothelial space promoting lesion initiation, which is usually followed by macrophage recruitment, VSMC migration and proliferation, fibrous cap formation, and atherosclerotic plaque development [65]. This process is generally stimulated by a combination of factors such as dyslipidemia, hyperglycemia, and shear stress that activate common pathways, promoting all the events leading to the development of atherosclerotic plaques. Interestingly, both OPG and TRAIL are found in atherosclerotic plaques [66], where they seem to participate in this process by exerting opposite actions (Figure 3).

As for OPG, the first studies evaluating its effects on the vasculature indicated that it could protect the vessels against calcification, given that OPG deficiency resulted in early-onset severe osteoporosis as well as significant medial calcification of the aorta and the arteries [67]. Similarly, OPG inactivation in ApoE-knockout mice resulted in augmented vascular calcification and increased size of atherosclerotic plaques, as compared to their controls [68]. However, in another study where LDLr-knockout mice were fed with an atherogenic diet and treated with fc-OPG, fc-OPG reduced plaque calcification but did not affect the number and size of the lesions, suggesting that although OPG protected against vascular calcification, it did not affect atherosclerosis progression and severity [69]. By contrast, our group has shown that human full-length OPG induced the proliferation of rodent vascular smooth muscle cells and increased atherosclerosis extension in diabetic ApoE-knockout mice, suggesting that this molecule could actually promote atherosclerosis [70]. Moreover, an infusion of full-length recombinant OPG in ApoE-knockout mice every 3 weeks for 3 months also resulted in increased vascular collagen content in the media [35].

To reconcile these results, it is possible that OPG, initially secreted to protect the vasculature against calcification, would actually damage it by promoting inflammation and fibrosis. The concept that OPG can actually promote atherosclerosis development is supported by several* in vitro* studies demonstrating that OPG has proinflammatory and profibrotic effects on the vasculature. As for inflammation, it has been demonstrated that when leukocyte-endothelial cell adhesion takes place, it increases the leukocyte production of proinflammatory cytokines such as TNF-*α* and interferon-*γ*, which would upregulate OPG expression in EC and VSMC [71–73]. Moreover, in line with the* in vitro* observation that OPG stimulates EC expression of adhesion molecules [73], we have recently shown that OPG increases leukocyte adhesion to endothelial cells [74] both* in vivo* and* in vitro*, contributing to atherosclerotic plaque formation. As for vascular fibrosis, consistent with our earlier finding that human full-length OPG induced the proliferation of rodent VSMC, we have found that VSMC treatment with full-length recombinant OPG induced fibrogenesis with increased expression of fibronectin, collagen I, collagen III, and collagen IV, as well as MMP-2 and MMP-9, and TGF-*β* [35]. Pretreatment with the specific TGF-*β* receptor inhibitor, prior to treatment with OPG, attenuated OPG-induced fibrogenesis and proliferation in VSMC. These results suggest that OPG is a potent inducer of fibrogenesis, growth factor synthesis, and proliferation in VSMC, both* in vitro* and* in vivo*, and that its actions are largely dependent on the autocrine induction of TGF-*β*, which itself stimulates OPG in a vicious cycle that results in the autoinduction of both OPG and TGF-*β* [35].

Nevertheless, OPG could also promote atherosclerosis by stimulating systemic inflammation and the renin-angiotensin system (RAS) activation, which is one of the most important pathways leading to atherosclerosis [75, 76]. As for systemic inflammation, we have recently shown that OPG delivery increases IL-6, MCP-1, and TNF-*α* circulating levels [77], which is consistent with the view that it takes part in the pathogenesis of atherosclerosis and CVD by amplifying inflammation [5]. Consistent with this claim, we have also reported a positive correlation between OPG and CRP [77]. With respect to the interplay with the RAS, experimental evidence suggests that there is a mutual stimulatory effect between OPG and the RAS [35, 78–82]. It has in fact been demonstrated that angiotensin II (Ang II) increases OPG expression in human aortic smooth muscle cells [78] as well as in murine VSMC [35]. Not surprisingly, treatment with the Ang II type 1 receptor (AT1R) blocker Irbesartan reduced OPG secretion from human abdominal aortic aneurysm explants [79]. Consistent with this finding, a recent study has demonstrated that AT1R blockade with Irbesartan significantly reduced OPG expression in human primary vascular cells and carotid atheromas [80]. Interestingly, if Ang II stimulates vascular OPG expression in a dose-dependent manner, OPG reciprocally stimulates vascular AT1R protein expression in a dose-dependent manner [81]. Consistent with this observation, we have observed that OPG delivery significantly increased ACE and AT1R gene and protein expression in the pancreas [82], where we hypothesized that OPG might control their transcription by activating the mitogen-activated protein kinase signaling [31] that regulates ACE and AT1R expression.

Interestingly, in addition to RAS blockers, there are other antiatherosclerotic drugs [83], such as statins and glitazones, which have exhibited the ability to reduce OPG in the vessels. As for statins, they reduced TNF-*α* and IL-1*α*-induced OPG expression in EC and VSMC [84]. As for glitazones, on the other hand, which are pharmacological PPAR-*γ* ligands, they significantly decreased the expression of OPG in human aortic smooth muscle cells [85].

### 3.2. TRAIL and Atherosclerosis

Contrary to OPG, animal studies [86–88] suggest that TRAIL protects against atherosclerosis. In the first of these studies, TRAIL treatment, delivered either as soluble recombinant TRAIL by intraperitoneal injection or in an adenoviral-vector, significantly reduced the accumulation and complexity of atherosclerotic plaques in diabetic ApoE-knockout mice [86]. Here, we speculated that TRAIL effects were mediated by its ability to induce apoptosis of infiltrating macrophages within the plaque, which had been previously observed* in vitro* by a different group [89]. The second study was conducted in TRAIL ApoE-double-knockout mice and demonstrated that TRAIL deficiency worsened atheromatous lesion formation, possibly by increasing VSMC content within the plaque [87]. In the mice lacking TRAIL, there was a reduction in VSMC apoptosis, indicating that TRAIL would induce VSMC apoptosis [90] rather than their survival [91] and that this could be the mechanism protecting against plaque enlargement. Consistent with our previous findings, Di Bartolo and colleagues reported a significant increase in atherosclerotic plaque formation and progression in ApoE- and TRAIL-double-knockout mice [88]. Here, TRAIL deficiency significantly influenced plaque stability, as it increased the extension of the necrotic core and macrophage infiltration, while reducing VSMC and collagen content [88]. This work is of particular interest not only because it confirms TRAIL antiatherosclerotic effects but also because it sheds light onto a possible role for TRAIL in glucose metabolism regulation [92]. Recently, it has also been shown that TRAIL inhibits vascular calcification [93], as TRAIL deficient mice exhibited a significant increase in tissue RANKL, which leads to vascular calcification. Consistent with this finding, VSMC exposed to calcium and TRAIL displayed significantly lower alizarin red staining (used to quantify vascular calcification) as compared to those exposed to calcium alone, indicating that TRAIL protects against calcium-induced VSMC calcification* in vitro* [93].

Overall, it is very difficult to draw conclusions on the mechanisms underlying the antiatherogenic effects of TRAIL by simply looking at* in vitro* data. Potentially, TRAIL is a molecule with two faces [94], the first that can induce apoptosis [95] and stimulate inflammation [45, 97] and the second that can promote cell survival [44, 96] and inhibit inflammation, depending on its dose and cell responsiveness. Nevertheless, animal studies show that TRAIL protects against atherosclerosis, possibly by inducing apoptosis of macrophages and VSMC [86–90]. Other potential mechanisms underlying TRAIL antiatherogenic effects include protection of normal vascular cells and anti-inflammatory actions [44, 92, 98, 99]. As mentioned earlier, both EC and VSMC express TRAIL receptors and Secchiero and colleagues have shown that recombinant TRAIL is able to promote their survival/proliferation by activating intracellular signaling pathways, such as ERK/MAPK, Akt, and NF-*κ*B, which are known to promote survival and proliferation [44]. Moreover, the same authors showed that TRAIL upregulates the production and release of prostanoids, including PGE2 and PGI2, and increases NO production and eNOS activity in endothelial cells, without activating NF-*κ*B, which are all involved in the maintenance of vascular homeostasis [98]. It has also been shown that TRAIL counteracts leukocyte adhesion induced by TNF-*α* or IL1-*β* by downregulation of CCL8 and CXCL10 chemokine expression [99]. This is consistent with the observation that TRAIL can significantly reduce systemic and tissue inflammation, as assessed by measuring IL-6, MCP-1, and TNF-*α* expression [92], which on the contrary were found elevated in TRAIL-knockout mice [88]. Recently, it has also been shown that administration of human recombinant TRAIL reduced allergic airway inflammation in a mouse model of asthma [100].

## 4. Clinical Applications of OPG and TRAIL as Biomarkers of CVD

### 4.1. OPG and CVD

Keeping in line with the dichotomy between the role of OPG and TRAIL in atherosclerosis (Figure 3), while TRAIL appears to be antiatherosclerotic, OPG has been shown to be associated with CVD onset and progression. OPG levels are in fact positively correlated with markers of vascular damage such as endothelial dysfunction [101–103], vascular stiffness [104], and coronary calcification [105], as well as with the presence of coronary artery disease (CAD) [106, 107]. Consistent with this, OPG has been found associated with the risk of future CAD in apparently healthy men and women, independent of established cardiovascular risk factors [8, 9]. In patients with acute coronary syndromes, OPG has been linked to the incidence of death, heart failure (HF) hospitalizations, myocardial infarction (MI), and stroke [108], which has been successively observed in the general population as well [109]. Moreover, although initially it appeared that OPG was an independent risk factor for incident CVD and vascular mortality but not for mortality due to nonvascular causes [8, 110], it has been recently demonstrated that high levels of OPG can also predict nonvascular mortality [111].

Left ventricular dysfunction is one of the key prognostic indicators of cardiovascular morbidity and mortality [112]. Interestingly, OPG has been found to be elevated in both clinical and experimental HF [10]. Moreover, different studies have evaluated the prognostic utility of OPG in patients with HF. In the first one, Ueland and colleagues showed that, in patients with history of myocardial infarction and left ventricular dysfunction, baseline OPG was significantly higher in those who died from vascular and nonvascular causes as compared to those who survived [113]. In a subsequent study, Omland and colleagues showed that in patients with acute coronary syndrome the baseline levels of OPG correlated significantly with the incidence of heart failure [108]. More recently it has been shown that OPG is predictive of hospitalization for HF in patients with advanced systolic HF and ischemic heart disease independently of conventional risk markers [114].

It is well known that diabetes mellitus and chronic kidney disease (CKD) are associated with an increased risk of CVD and vascular mortality [115, 116]. Interestingly, in both conditions OPG levels are elevated and predict CVD onset. Several groups have reported that OPG levels are elevated in patients with type 1 and type 2 DM, as reviewed in [6]. Nevertheless, beside the positive relationship between OPG and type 2 DM, which has been known since 2001 [117], in diabetic patients there is also a strong association between circulating levels of OPG and micro- and macrovascular complications [118, 119]. Here, OPG is associated with cardiovascular events [119, 120] and the presence and severity of silent myocardial ischemia [121–124], as well as with the risk of developing end-stage renal disease [125]. Consistent with the experimental data showing an inhibitory effect of glitazones on vascular OPG [85], in type 2 DM patients, pioglitazone was found to decrease OPG levels [126, 127], which showed correlation with glucose control [126].

As for CKD, on the other hand, OPG is increased in patients with nondiabetic [128, 129] and diabetic [119, 125, 130] CKD, where it predicts kidney function deterioration and vascular events and cardiovascular and all-cause mortality [130]. Consistent with implications in CKD, it has been recently reported that elevated OPG is associated with increased 5- and 10-year risk of rapid renal decline, renal disease hospitalization, and/or deaths in elderly women [131].

### 4.2. TRAIL and CVD

Contrary to OPG, the serum levels of TRAIL have been found significantly decreased in patients affected by or predisposed to CVD. In regard to this issue, it is notable that serum levels of TRAIL are significantly decreased in patients with acute myocardial infarction within 24 hours of admission, compared to healthy controls [132]. Relatedly, also Michowitz and colleagues found that circulating TRAIL was significantly lower in patients with acute coronary syndrome as compared to those with stable angina or normal coronary arteries and that it was negatively correlated with the level of C-reactive protein, which is an independent predictor of acute vascular events and adverse outcomes in patients with HF [133]. Given that the same authors found that TRAIL expression was increased in vulnerable plaques, where it localized with T cells and oxidized low-density lipoprotein, they argued that TRAIL decrease in patients with CVD might be due to its consumption into the plaques. Other reasons underlying TRAIL decrease in patients with acute cardiovascular events might include the parallel increase in circulating OPG, as well as the increase of metalloproteinase-2 (MMP-2). While OPG acts as a decoy receptor for TRAIL, whereby its binding may interfere with TRAIL dosage explaining TRAIL decrease, the increase in MMP2 could explain TRAIL decrease as it has been shown that MMP-2 can induce TRAIL cleavage [134].

Consistent with these findings, circulating TRAIL levels are inversely associated with an increased risk of CVD and cardiac mortality [132, 135]. In the work by Secchiero and colleagues the patients with myocardial infarction who developed in-hospital adverse clinical outcomes displayed the lowest levels of TRAIL, indicating that the lower the level of TRAIL, the higher the risk of HF or death after myocardial infarction [132]. In the work by Michowitz and colleagues low TRAIL levels at discharge were associated with an increased incidence of cardiac death and heart failure in the 1-year follow-up [133]. Similarly, an inverse association of TRAIL levels with mortality was observed in patients with advanced heart failure [136], as well as in patients with CKD [137]. Moreover, in older patients (i.e., aged on average 68 years) with cardiovascular diseases, low levels of TRAIL were associated with increased risk of death over a period of 6 years [135].

## 5. Conclusions

Experimental studies suggest that there is some dichotomy in OPG and TRAIL actions, the first being proatherogenic and the second being antiatherogenic. However, the role of OPG and TRAIL in atherosclerosis has not been fully understood yet. It remains unclear whether OPG increase and TRAIL decrease should be regarded as risk factors rather than risk markers of CVD; therefore, further studies are needed to clarify what the pathogenic importance of OPG and TRAIL is in the process of atherosclerosis. On the other hand, clinical studies reinforce the view that OPG and TRAIL could be promising biomarkers of CVD onset and progression. More evidence (possibly gained after measurement standardization) is needed to evaluate the predictive and diagnostic value of OPG and TRAIL for clinical use.
